# Multicenter Belgian prospective registry on minimally invasive and open liver surgery (BReLLS): experience from 1342 consecutive cases

**DOI:** 10.1007/s00423-025-03661-4

**Published:** 2025-03-03

**Authors:** Roberto Ivan Troisi, Gianluca Rompianesi, Mathieu D’Hondt, Aude Vanlander, Claude Bertrand, Catherine Hubert, Olivier Detry, Bert Van den Bossche, Philippe Malvaux, Joseph Weerts, Thibault Sablon, Koen Vermeiren, Mehrdad Biglari, Filip Gryspeerdt, Celine De Meyere, Alexandra Dili, Kim Boterbergh, Valerio Lucidi

**Affiliations:** 1 Faculty of Medicine, Ghent University, Ghent, Belgium; 2 Federico II University Hospital, via S. Pansini 5, 80131 Naples, Italy; 3 AZ Groeninge Kortrijk, Kortrijk, Belgium; 4 VUB Jette Brussels, Jette, Belgium; 5 CHU-UCL Namur - site Mont Godinne, Yvoir, Belgium; 6 UCL - Saint Luc– Brussels, Brussels, Belgium; 7 Department of Abdominal Surgery and Transplantation - CHU Liege, University of Liege, Liège, Belgium; 8 ASZ Aalst, Aalst, Belgium; 9 Centre Hospitalier de Wallonie Picard site Notre-Dame, Tournai, Belgium; 10 Clinique Saint-Joseph, Liege, Belgium; 11 VITAZ Sint-Niklaas, Sint-Niklaas, Belgium; 12 Imelda Ziekenhuis, Bonheiden, Belgium; 13 Sint-Andries Ziekenhuis, Tielt, Belgium; 14 Heilig Hart Ziekenhuis, Liers, Belgium; 15 Universite Libre de Bruxelles– Erasme Hospital, Brussels, Belgium

**Keywords:** Liver surgery, Minimally invasive surgery, Laparoscopic surgery, Open surgery, Outcomes

## Abstract

**Purpose:**

Minimally invasive liver surgery (MILS) still appears to be adopted with significant variability. We aimed to investigate the diffusion, indications, and short-term outcomes of MILS compared to the open approach.

**Methods:**

A prospective registry of all liver resections performed for any indication and using any technique between January 1, 2017, and December 31, 2019, was established (BReLLS) and analyzed.

**Results:**

A total of 1342 consecutive liver resections were included, 684 (51%) MILS and 658 (49%) open procedures. MILS was not attempted due to technical complexity in the 46.2% of cases, followed by previous abdominal surgery (22.5%). Patients undergoing MILS had a higher proportion of benign indications and of hepatocellular carcinomas, patients affected by liver cirrhosis with portal hypertension and a lower proportion of major hepatectomies (all *p* < 0.001). After propensity-score matching, MILS showed better results in terms of surgery duration (*p* < 0.001), blood loss (*p* = 0.015), complication rate (*p* < 0.001), rate of Clavien-Dindo grade ≥ 3 complications (*p* = 0.012), comprehensive complication index (*p* < 0.001), length of stay (*p* < 0.001), readmissions (*p* = 0.016). Centers performing over 50 liver resections per year had a higher proportion of overall MILS cases (*p* < 0.001), a similar proportion of major resections (*p* = 0.362), but a higher prevalence of MILS major resections (*p* = 0.004), lower 90-day mortality rates (*p* < 0.001), lower overall complication rates (*p* < 0.001), and shorter hospital length of stay (*p* < 0.001).

**Conclusion:**

MILS was the preferred technique in half of the cases, particularly in patients with cirrhosis and portal hypertension, and benign lesions. It provided superior short-term outcomes compared to the open approach for both minor and major liver resections in selected patients.

## Introduction

Hepatobiliary surgery is a rapidly developing surgical specialty and represents the treatment of choice for various benign and malignant conditions [1–4]. Major advances have been introduced in this field over the past decades, with one of the most impactful being the progressive adoption of minimally invasive techniques, including the robotic-assisted approach [5–8]. There is growing evidence and consensus on the benefits of minimally invasive liver surgery (MILS) for treating various benign and malignant conditions, as well as for living-donor hepatectomies [9–13].

MILS has been shown to offer advantages in terms of fewer postoperative complications, reduced liver decompensation in cirrhotic patients, earlier functional recovery, shorter hospital stays, and fewer readmissions, while still ensuring optimal oncological outcomes [12, 14]. These benefits consequently translate into reduced resource utilization and lower healthcare costs [15]. Minimally invasive techniques are no longer restricted to easily accessible tumors. Resections that were once considered complex due to lesion size, location, or extent are now performed with good results. However, such procedures remain challenging and should be undertaken with caution by experienced surgeons [14]. According to a recent European survey, MILS was performed with a median annual proportion of 30% per center (16–40%), with the robotic technique being utilized in one-third of the centers. These figures are expected to increase over time [16]. This variability is also reflected in the absolute number of MILS procedures performed annually at each center, translating into poorer outcomes in low-volume programs, similar to what has been previously described for open liver resections [17, 18].

The need to generate high-quality evidence to fully understand the limitations and advantages of MILS compared to the open approach is hindered by the ethical and practical constraints of conducting randomized controlled trials, as well as the lack of national and international registries that include consecutive minimally invasive and open liver resections. The **BReLLS** (Belgian Registry on Laparoscopic Liver Surgery) project is a prospective, non-compulsory, multicentric online registry of MILS and open liver procedures. It was initiated in 2016 and is endorsed by the Belgian Section of Hepato-Biliary and Pancreatic Surgery. The primary aim of this project is to monitor the evolution and adoption of MILS in Belgium. The secondary aim is to evaluate and compare the short-term outcomes of MILS and open liver resections.

## Materials and methods

Thirteen Belgian divisions of general and hepatobiliary surgery participated in this project between January 1, 2017, and December 31, 2019, to establish a prospective, non-compulsory online registry of consecutive minimally invasive and open liver procedures (BReLLS). The participating centers were: Ghent University Hospital (Ghent); AZ Groeninge (Kortrijk); UCL - Saint Luc (Brussels); Free University Erasme (Brussels); CHU-Namur– Mont Godinne; University Hospital Center of Liege; ASZ Aalst; Centre Notre-Dame Hospital (Tournai); Cliniques Saint-Joseph (Liège); AZ Nikolaas; Imelda Ziekenhuis (Bonheiden); Sint-Andries Hospital (Tielt); and Heilig Hart Ziekenhuis (Leuven). Demographic, radiologic, clinical, surgical, pathological, and follow-up data were collected through a Secure Sockets Layer (SSL) server (www.brells.org) from prospectively maintained databases. All consecutive patients undergoing liver surgery for any indication and using any technique were included. The BRELLS steering committee carried out random checks in the participating centres to verify the correctness of the data entered on the platform. Liver resections were classified according to the Brisbane terminology [19]. Major liver resections were defined as the excision of 3 or more liver segments. he complexity of surgery was graded according to Ghent [20] and IWATE [21] difficulty scores. Readmissions were defined as the need for hospitalization following discharge.

The primary endpoint was to assess the adoption, indications, and factors limiting the use of MILS in Belgium. The secondary endpoints included comparisons of surgical outcomes and short-term results between the minimally invasive and open approaches.

### Statistical analysis

Descriptive statistics for all collected variables have been performed. The distribution characteristic of the measured data was analysed with the Shapiro-Wilk test and the homogeneity test of variances. Normally distributed quantitative variables have been reported as mean values and standard deviation (SD) and compared using the two-sided Student’s t-test, otherwise as median and range and compared using the Mann–Whitney test. Qualitative variables are expressed as counts and percentages and compared using the Chi-Squared test with Yates’ correction or the Fisher’s exact test.

Propensity score matching was applied to reduce selection bias between groups. The propensity score was estimated through a logistic regression model, including baseline variables potentially influencing the outcomes, including patients’ age, sex, BMI, ASA score, presence of cirrhosis and portal hypertension, presence of a malignant tumor, presence of previous abdominal surgery, number and size of liver lesions, major or minor resection, and IWATE difficulty score. Patients receiving MILS were then 1:1 matched to patients undergoing open surgery using nearest-neighbor matching without replacement or discard, using a caliper of 0.2. Patients with missing data in any of the variables used for matching were discarded.

P-values < 0.05 have been considered statistically significant. All tests have been two-sided. The data analyses have been performed with SPSS (version 28, IBM, New York, NY, USA) and MedCalc (Software for Windows, Version 14.8.1, Ostend, Belgium).

## Results

A total of 1,530 liver-directed procedures were reported in the database. After excluding liver cyst fenestration and ablation-only cases (188 patients, 12.3%), 1,342 consecutive liver resections were included in the BReLLS database, consisting of 684 (51%) minimally invasive liver surgeries (MILS) and 658 (49%) open procedures (Table 1). MILS was performed laparoscopically in 662 cases (96.8%), robotically assisted in 13 cases (1.9%), and hand-assisted in 9 cases (1.3%). The primary reasons for not attempting a MILS approach were technical complexity (46.2%), previous abdominal surgery (22.5%), and the necessity of performing associated procedures (9.9%) (Table 2).

**Table 1:** Minimally invasive and open liver resections: patient demographics. MILS, minimally invasive liver surgery; BMI, body mass index; ASA, American society of anaesthesiologists; CRLM, colorectal liver metastases; HCC, hepatocellular carcinoma; CCC, cholangiocarcinoma; CA, adenocarcinoma; NET, neuroendocrine tumour, FNH, focal nodular hyperplasia; NA, not available; SD, standard deviation. *, calculated on malignant cases only

	MILS *N* = 684 (51.0%)	Open *N* = 658 (49.0%)	*p*
Age, years	62.4 ± 13.7	62.3 ± 13.7	0.894
Gender (M/F), n (%)	412 (60.2%) / 272 (39.8%)	396 (60.2%) / 262 (39.8%)	0.985
BMI	26.7 ± 5.1	26.1 ± 5.1	**0.0314**
ASA score	2.2 ± 0.62	2.2 ± 0.62	> 0.999
Benign/Malignant	120 (17.5%) / 564 (82.5%)	72 (10.9%) / 586 (89.1%)	**< 0.001**
Indication, n (%)			
CRLM HCC CCC Non-colorectal metastases Gallbladder CA NET Adenoma FNH Haemangioma Other Unknown	292 (42.7%) 172 (25.1%) 21 (3.1%) 21 (3.1%) 6 (0.9%) 8 (1.2%) 37 (5.4%) 16 (2.3%) 9 (1.3%) 78 (11.4%) 24 (3.5%)	356 (54.1%) 76 (11.6%) 67 (10.2%) 11 (1.7%) 21 (3.2%) 9 (1.4%) 7 (1.1%) 6 (0.9%) 3 (0.5%) 87 (13.2%) 15 (2.3%)	**< 0.001**
Previous abdominal surgery	366 (53.5%)	427 (64.9%)	**< 0.001**
Previous liver surgery	89 (13.0%)	172 (26.1%)	**< 0.001**
Liver parenchyma, n (%)			
Normal Steatosis Fibrosis Cirrhosis NA	358 (52.3%) 126 (18.4%) 30 (4.4%) 128 (18.7%) 42 (6.1%)	399 (60.6%) 140 (21.3%) 33 (5.0%) 52 (7.9%) 34 (5.2%)	**< 0.001**
Cirrhosis etiology, n (%)			
Alcohol Viral NASH Cryptogenic NA	65 (50.8%) 33 (25.8%) 23 (17.9%) 2 (1.6%) 5 (3.9%)	29 (55.8%) 18 (34.6%) 3 (5.8%) 2 (3.8%) 0	0.182
Portal hypertension	67 (9.8%)	23 (3.5%)	**< 0.001**
Number of lesions, median (range)	1 (1–19)	1 (1–33)	**< 0.001**
Diameter of the largest lesion, average ± SD (mm)	34.0 ± 39.6	47.1 ± 39.6	**< 0.001**
Preoperative chemotherapy*	184 (36.9%)	324 (60.0%)	**< 0.001**

**Table 2:** Minimally invasive and open liver resection operative characteristics comparison. MILS, minimally invasive liver surgery; SD, standard deviation. *, calculated on malignant cases only

	MILS *N* = 684 (51.0%)	Open *N* = 658 (49.0%)	*p*
MILS Technique, n (%)			-
Laparoscopic Robotic Hand-assisted	662 (96.8%) 13 (1.9%) 9 (1.3%)	- - -	
Reason precluding MILS, n (%)	-		-
Technical/oncological complexity Previous abdominal surgery Combined procedures Surgeon’s individual choice Part of a trial (randomized open) Others Unreported		304 (46.2%) 148 (22.5%) 65 (9.9%) 31 (4.7%) 14 (2.1%) 27 (4.1%) 69 (10.5%)	
Major resections, n (%)	67 (9.8%)	244 (37.1%)	**< 0.001**
Brisbane classification, n (%)			**< 0.001**
Right hepatectomy Left hepatectomy Right anterior sectionectomy Right posterior sectionectomy Left medial sectionectomy Left lateral sectionectomy Right trisectionectomy Left trisectionectomy Segmentectomy Bisegmentectomy Unknown	31 (4.5%) 26 (3.8%) 11 (1.6%) 41 (6.0%) 5 (0.7%) 75 (11.0%) 5 (0.7%) 5 (0.7%) 215 (31.4%) 48 (7.0%) 222 (32.5%)	94 (14.3%) 68 (10.3%) 11 (1.7%) 22 (3.3%) 12 (1.8%) 21 (3.2%) 38 (5.8%) 12 (1.8%) 145 (22.0%) 62 (9.4%) 173 (26.3%)	
Postero-superior segments resections, n (%)	93 (13.6%)	79 (12.0%)	0.384
Ghent difficulty score, average ± SD	4.47 ± 3.05	5.76 ± 3.05	**< 0.001**
IWATE score, average ± SD	5.52 ± 2.48	6.97 ± 2.48	**< 0.001**
Pringle manoeuvre, n (%)	241 (35.2%)	259 (39.4%)	0.118
Pringle duration, average ± SD (min)	37.5 ± 24.3	29.0 ± 22.2	**< 0.001**
Blood loss, average ± SD (ml)	284 ± 735	507 ± 735	**< 0.001**
Abdominal drainage, n (%)	397 (58.0%)	587 (89.2%)	**< 0.001**
Surgery duration, average ± SD (min)	185 ± 126	270 ± 127	**< 0.001**
Conversion, n (%) Open Hand assisted	53 (7.7%) 47 (6.9%) 6 (0.9%)	-	
Reason Conversion, n (%)		-	-
Bleeding Oncologic/anatomic reason Adhesions Other/non stated	21 (39.6%) 15 (28.3%) 11 (20.8%) 6 (11.3%)		
Specimen margins*, n (%)			**0.006**
R0 R1 R2 Unknown	421 (74.6%) 63 (11.2%) 2 (0.4%) 78 (13.8%)	424 (72.4%) 95 (16.2%) 8 (1.4%) 59 (10.1%)	

Patients undergoing MILS had a higher average body mass index (BMI, 26.7 ± 5.1 vs. 26.1 ± 5.1, *p* = 0.0314), a significantly higher proportion of benign indications (17.5% vs. 10.9%, *p* < 0.001) and hepatocellular carcinoma (HCC, 25.1% vs. 11.6%, *p* < 0.001). They were less likely to have had previous abdominal (53.5% vs. 64.9%, *p* < 0.001) and liver surgeries (13.0% vs. 26.1%, *p* < 0.001) and had fewer and smaller liver lesions (1 [1–19] and 34.0 ± 39.6 mm vs. 1 [1–33] and 47.1 ± 39.6 mm, *p* < 0.001). Additionally, a significantly higher proportion of patients with liver cirrhosis (18.7% vs. 7.9%, *p* < 0.001) and portal hypertension (9.8% vs. 3.5%, *p* < 0.001) underwent MILS. The complete patient demographic data are shown in Table 1.

### Operative results

Patients undergoing MILS had a significantly lower proportion of major hepatectomies (9.8% vs. 32.2%, *p* < 0.001). The most frequently performed resections in this group were segmentectomies (31.4%), left lateral sectionectomies (11.0%), and bi-segmentectomies (7.0%), compared to segmentectomies or non-anatomical resections (22.0%), right hepatectomies (14.3%), and left hepatectomies (10.3%) in patients undergoing open surgery (*p* < 0.001, Table 2). There was no significant difference in the posterosuperior segment resection rate (13.6% vs. 12.0%, *p* = 0.384). However, both the average Ghent [20] (4.47 ± 3.05 vs. 5.76 ± 3.05, *p* < 0.001) and IWATE [21] (5.52 ± 2.48 vs. 6.97 ± 2.48, *p* < 0.001) difficulty scores were lower in the MILS group.

### Postoperative outcomes

MILS patients demonstrated higher R0 resection rates (*p* = 0.006) and better postoperative outcomes, including shorter hospital stays, lower overall complication rates, fewer Clavien-Dindo grade ≥ 3 complications, lower comprehensive complication index (CCI), fewer readmissions, and lower 90-day mortality (all *p* < 0.001). Among the 23 recorded 90-day mortalities, cardiovascular complications were the leading cause (47.8%), followed by sepsis (21.7%) and pulmonary complications (13.0%).

The better post-operative outcomes in patients undergoing MILS were confirmed in all evaluated parameters for patients undergoing minor liver resections, and only in terms of shorter hospital length of stay, lower complication rate, and lower CCI in patients undergoing major liver resections (Tables 2 and 3).

**Table 3:** Minimally invasive and open liver resections: perioperative outcomes. MILS, minimally invasive liver surgery; SD, standard deviation; CCI, comprehensive complication index

	MILS *N* = 684 (51.0%)	Open *N* = 658 (49.0%)	*p*
Hospital length of stay, average ± SD (days)	5.2 ± 7.0	9.7 ± 7.0	**< 0.001**
Complications (overall), n (%)	212 (31.0%)	357 (54.3%)	**< 0.001**
CCI, average ± SD	5.0 ± 11.5	14.2 ± 20.5	**< 0.001**
Complications Clavien-Dindo grade ≥ 3	26 (3.8%)	106 (16.1%)	**< 0.001**
Readmissions, n (%)	36 (5.3%)	75 (11.4%)	**< 0.001**
90-day mortality, n (%)	3 (0.4%)	20 (3.0%)	**< 0.001**
Major Resections	*N* = 67 (9.8%)	*N* = 244 (37.1%)	
Hospital length of stay, average ± SD (days)	7.8 ± 7.0	11.6 ± 9.5	**< 0.001**
Complications (overall), n (%)	23 (34.3%)	150 (61.5%)	**< 0.001**
CCI, average ± SD	9.4 ± 18.9	18.7 ± 24.3	**< 0.001**
Complications Clavien-Dindo grade ≥ 3, n (%)	7 (10.4%)	51 (20.9%)	0.052
Readmissions, n (%)	5 (7.5%)	34 (13.9%)	0.156
90-day mortality, n (%)	1 (1.5%)	13 (5.3%)	0.180
Minor Resections	*N* = 617 (90.2%)	*N* = 414 (62.9%)	
Hospital length of stay, average ± SD (days)	4.9 ± 5.2	8.6 ± 6.7	**< 0.001**
Complications (overall), n (%)	189 (30.6%)	207 (50.0%)	**< 0.001**
CCI, average ± SD	4.5 ± 10.2	11.6 ± 17.4	**< 0.001**
Complications Clavien-Dindo grade ≥ 3, n (%)	19 (3.1%)	55 (13.3%)	**< 0.001**
Readmissions, n (%)	31 (5.0%)	41 (9.9%)	**0.003**
90-day mortality, n (%)	2 (0.3%)	7 (1.7%)	**0.021**

### Results according to center volume

**Fig. 1:**
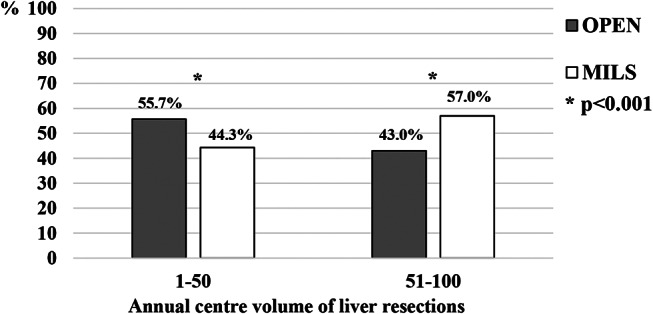
Proportions of open and minimally invasive liver resections in centres performing up to 50 and over 50 liver resections per year. MILS, minimally invasive liver surgery

**Fig. 2:**
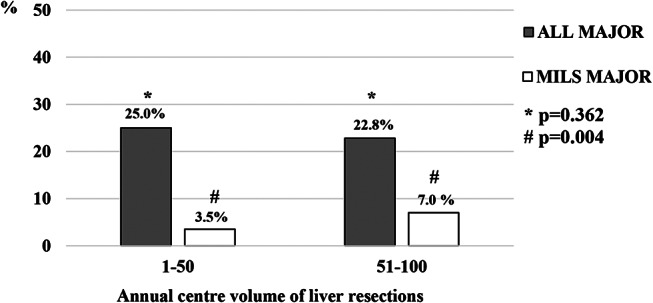
Proportions of major liver resections and minimally invasive major liver resections in centres performing up to 50 and over 50 liver resections per year. MILS, minimally invasive liver surgery

Three centers (23.1%) achieved a yearly volume of 50 or more liver resections, accounting for 705 out of 1,342 resections (52.5%). High-volume centers performed MILS in 57.0% of cases, compared to 44.3% of patients in low-volume centers (*p* < 0.001), had a lower conversion rate (4.7% vs. 12.3%, *p* < 0.001) with a similar proportion of major liver resections (*p* = 0.362) but 2-time higher prevalence of MILS major resections (*p* = 0.004, Figs. 1 and 2). High-volume centers performed cases with a higher average IWATE difficulty score (6.2 ± 2.5 vs. 5.4 ± 2.5, *p* < 0.001). Patients undergoing liver resections in high-volume centers had lower 90-day mortality rates (1.0% vs. 2.5%, *p* < 0.001), lower overall complication rates (24.2% vs. 54.9%, *p* < 0.001), lower although borderline-significant Clavien-Dindo grade ≥ 3 complications (8.4% vs. 11.5%, *p* = 0.058), and a shorter hospital length of stay (6.4 ± 7.0 vs. 8.6 ± 7.0 days, *p* < 0.001).

### Propensity score matching analysis

A 1:1 matching of MILS versus open liver resections was performed in order to avoid the impact of confounders, obtaining two fully comparable groups according to baseline patients, liver lesions and surgical characteristics. After matching, the two groups consisted of 303 patients each, the 45.2% of the whole cohort. The postoperative short-term outcomes of MILS remained superior to open liver resections in terms of duration of surgery (*p* < 0.001), intraoperative blood loss (*p* = 0.015), hospital LOS (*p* < 0.001), overall complication rate (*p* < 0.001), rate of Clavien-Dindo grade ≥ 3 complications (*p* = 0.012), CCI (*p* < 0.001), and readmission rate (*p* = 0.016), with no difference in terms of R0 resection rates (*p* = 0.861) and 90-day mortality (*p* = 0.254). The patients undergoing major resections in the MILS and open groups were 47 (15.5%) and 64 (19.8%), respectively, with MILS resulting superior in terms of intraoperative blood loss (*p* = 0.048), hospital LOS (*p* < 0.001), overall complication rate (*p* = 0.016), and CCI (*p* = 0.001). In minor liver resections MILS resulted superior in terms of duration of surgery (*p* < 0.001), hospital LOS (*p* < 0.001), overall complication rate (*p* < 0.001), rate of Clavien-Dindo grade ≥ 3 complications (*p* = 0.015), CCI (*p* < 0.001), and readmissions (*p* = 0.027) as depicted in Table 4.

**Table 4:** Minimally invasive and open liver resections: perioperative outcomes following propensity score matching MILS, minimally invasive liver surgery; SD, standard deviation; CCI, comprehensive complication index

Propensity Score Matching			
	MILS *N* = 303	Open *N* = 303	*p*
All Resections			
Surgery duration, average ± SD (min)	194 ± 99	253 ± 133	**< 0.001**
Blood loss, average ± SD (ml)	331 ± 554	467 ± 795	**0.015**
Specimen margins*, n(%)			
R0 R1 R2 Unknown	231 (76.2%) 40 (13.2%) 2 (0.7%) 5 (1.7%)	230 (75.9%) 42 (13.9%) 3 (1.0%) 3 (1.0%)	0.861
Hospital length of stay, average ± SD (days)	5.5 ± 5.6	8.8 ± 7.2	**< 0.001**
Complications (overall), n(%)	75 (24.3%)	139 (45.9%)	**< 0.001**
CCI, average ± SD	5.8 ± 13.6	11.6 ± 6.5	**< 0.001**
Complications Clavien-Dindo grade ≥ 3, n(%)	19 (6.3%)	37 (12.2%)	**0.012**
Readmissions, n(%)	19 (6.3%)	36 (11.9%)	**0.016**
90-day mortality, n(%)	2 (0.7%)	5 (1.7%)	0.254
Major Resections	*N* = 47 (15.5%)	*N* = 64 (19.8%)	
Surgery duration, average ± SD (min)	281 ± 100	288 ± 130	0.759
Blood loss, average ± SD (ml)	544 ± 557	820 ± 819	**0.048**
Specimen margins*, n(%)			
R0 R1 R2 Unknown	39 (88.6%) 4 (9.1%) 1 (2.3%) 0	51 (89.5%) 4 (7.0%) 2 (3.5%) 0	0.876
Hospital length of stay, average ± SD (days)	7.9 ± 5.7	13.0 ± 7.2	**< 0.001**
Complications (overall), n(%)	17 (36.2%)	38 (59.4%)	**0.016**
CCI, average ± SD	11.6 ± 4.9	20.4 ± 17.6	**0.001**
Complications Clavien-Dindo grade ≥ 3, n(%)	7 (14.9%)	12 (18.8%)	0.594
Readmissions, n(%)	5 (10.6%)	10 (15.6%)	0.448
90-day mortality, n(%)	1 (2.1%)	4 (6.3%)	0.334
Minor Resections	*N* = 256 (84.5%)	*N* = 239 (80.2%)	
Surgery duration, average ± SD (min)	177 ± 97	244 ± 133	**< 0.001**
Blood loss, average ± SD (ml)	292 ± 551	375 ± 795	0.175
Specimen margins*, n(%)			
R0 R1 R2 Unknown	192 (81.4%) 36 (15.3%) 1 (0.4%) 5 (2.1%)	179 (81.0%) 38 (17.2%) 1 (0.5%) 3 (1.4%)	0.887
Hospital length of stay, average ± SD (days)	4.9 ± 5.6	7.8 ± 7.2	**< 0.001**
Complications (overall), n(%)	58 (22.7%)	101 (42.3%)	**< 0.001**
CCI, average ± SD	4.7 ± 13.7	9.4 ± 17.1	**< 0.001**
Complications Clavien-Dindo grade ≥ 3, n(%)	12 (4.7%)	25 (10.5%)	**0.015**
Readmissions, n(%)	14 (5.5%)	26 (10.9%)	**0.027**
90-day mortality, n(%)	1 (0.4%)	1 (0.4%)	0.961

*calculated on malignant cases only

### Utilization of surgical devices

In MILS a median of 4 trocars (3–7) have been used, 3 (3–7) for minor resections and 5 (4–7) for major resections. In terms of instruments and devices, the argon plasma coagulator (12.9% vs. 34.8%, *p* < 0.001), bipolar coagulation (31.1% vs. 42.6%, *p* < 0.001), cavitron ultrasonic surgical aspirator (40.9% vs. 70.4%, *p* < 0.001), and haemostatic agents or patches (75.1% vs. 81.2%, *p* = 0.008) have been utilized more often in open liver resections, as opposed to staplers (30.0% vs. 21.9%, *p* < 0.001) and energy devices (86.0% vs. 46.0%, *p* < 0.001), that have been utilized more often in MILS. The most frequently utilized energy device was the LigaSure™ (41.8% vs. 24.9%, *p* < 0.001), followed by the Thunderbeat™ (29.3% vs. 14.6%, *p* < 0.001), and the Ultracision Harmonic scalpel™ (14.9% vs. 7.0%, *p* < 0.001) in both MILS and open surgery, respectively (Table 5).

**Table 5:** Disposable instruments and devices utilization in minimally invasive and open surgical procedures. MILS, minimally invasive liver surgery; SD, standard deviation

	MILS *N* = 684 (51.0%)	Open *N* = 658 (49.0%)	*p*
Trocars number, median (range)			
All	4 (3–7)	-	-
Major resections	5 (4–7)	-	-
Minor resections	3 (3–7)	-	-
Retrieval bag	497 (72.7%)	-	-
Major resections	45 (67.2%)	-	-
Minor resections	452 (73.3%)	-	-
Argon plasma coagulator	88 (12.9%)	229 (34.8%)	**< 0.001**
Bipolar coagulation	213 (31.1%)	280 (42.6%)	**< 0.001**
Cavitron ultrasonic surgical aspirator	280 (40.9%)	463 (70.4%)	**< 0.001**
Energy devices	588 (86.0%)	303 (46.0%)	**< 0.001**
LigaSure™	286 (41.8%)	164 (24.9%)	**< 0.001**
Thunderbeat™	200 (29.3%)	93 (14.6%)	**< 0.001**
Ultracision Harmonic scalpel™	102 (14.9%)	46 (7.0%)	**< 0.001**
Staplers	205 (30.0%)	144 (21.9%)	**< 0.001**
Number used, average ± SD	2.95 ± 2.1	2.28 ± 2.1	**< 0.001**
Haemostatic agents/patches	514 (75.1%)	534 (81.2%)	**0.008**

## Discussion

This is the first report from a multicentric prospective registry providing results on consecutive liver resections over a three-year timeframe, including both open and minimally invasive techniques, offering valuable insights into their prevalence, applications, and indications. The BReLLS registry originates from the outcomes of the International Consensus Conference held in 2014 in Morioka, which emphasized the importance of obtaining high-level evidence on outcomes and mortality following major and minor minimally invasive and open liver surgeries through the establishment of registries [11].

Since the first consensus meeting in 2008, where acceptable indications for laparoscopic liver resections were limited to solitary lesions < 5 cm not located in posterosuperior segments and where only minor resections were considered standard practice [10], MILS has progressively addressed initial concerns regarding oncological safety, cost, and its relatively long learning curve. It has now established itself as a viable option for various malignant and benign indications, with the potential to be safely adopted in complex scenarios such as reoperations, challenging segments, major resections, and living-donor hepatectomies [12, 22].

Despite these advancements, MILS is still predominantly used for minor liver resections and less than 20% of major hepatectomies [16]. Moreover, a recent global liver surgery survey revealed that more than one-third of respondents had never performed major minimally invasive liver resections [23]. Significant regional and inter-center variations were observed in a pan-European survey, where MILS accounted for approximately one-third of liver resections on average but ranged from 0 to 100% (51% in this series), with annual volumes varying from a few cases to several hundred [16]. Interestingly, an inverse correlation was noted between the number of liver resections performed and the proportion of MILS.

The predominant MILS technique among the centers included in the registry was laparoscopic (96.8%), with only one center performing robotic liver resections during the study period, representing just 1.9% of total cases (Table 2).

According to our findings, MILS appears to be the preferred technique for resecting HCC in patients with cirrhosis and portal hypertension (Table 1), as it aims to minimize postoperative liver impairment and facilitate prompt recovery [24–27]. Although CRLM was the most common indication for both groups, it was more frequently managed with the open approach (54.1% vs. 42.7%), likely due to the number of lesions, the need for repeat liver resections, and the context of parenchyma-sparing surgery [28]. The lower proportion of CRLM patients receiving neoadjuvant chemotherapy in the MILS group (36.9%) may reflect an upfront surgery strategy for single lesions or oligometastatic disease, as per local center policies. This is further supported by the lower average number and diameter of lesions in the MILS group. Our results confirmed MILS as a viable option for the surgical management of benign liver lesions, accounting for 62.5% of cases (*p* < 0.001), aligning with existing literature that reports increasing use of minimally invasive techniques for small or undefined nodules [29, 30]. In recent years, robotic-assisted liver surgery has expanded rapidly, closing the gap with laparoscopy in terms of the number of centers adopting this technique and the volume of cases performed [31]. One advantage driving the spread of robotic liver surgery is its significantly shorter learning curve, potentially requiring only half the time needed for laparoscopy [32, 33]. Notably, more complex surgeries may benefit from this approach, yielding superior perioperative results compared to laparoscopy [34]. Unfortunately, during the BReLLS registry timeframe (2017–2019), only a few robotic cases were performed in Belgium, making a meaningful benefit analysis unfeasible.

On the other hand, the open approach was preferred in complex cases. In 46.2% of cases where MILS was not adopted, the primary reasons included the need for hepatojejunostomy or achieving satisfactory oncological results for large, multiple, or lesions adjacent to major vascular structures. The lowest percentage of MILS was observed in patients with adenocarcinoma of the gallbladder. This could be explained by both the technical complexity of performing a radical lymphadenectomy and the proximity or involvement of hilar structures in more advanced stages (i.e. IIIA and beyond). The higher complexity of patients undergoing open surgery was further supported by the significantly higher average Ghent and IWATE scores (both *p* < 0.001), longer average operative time (*p* < 0.001), and a fourfold higher rate of major resections (32.2% vs. 9.8%, *p* < 0.001).

Our findings demonstrated favorable postoperative outcomes, with a 90-day mortality rate below 2% and Clavien-Dindo grade ≥ 3 complications occurring in 9.8% of cases (Table 3). All evaluated outcome results were comparable to those reported in other large series [35–37]. Although MILS was generally performed in lower-complexity cases, it was superior to open surgery in terms of reduced hospital length of stay, overall complication rates, Clavien-Dindo grade ≥ 3 complications, CCI, readmission rates, and 90-day mortality (all *p* < 0.001). These findings were consistent in patients undergoing minor resections and, for hospital length of stay, overall complications, and Clavien-Dindo grade ≥ 3 complications, in patients undergoing major resections (Table 3). We controlled for potential confounding factors using propensity score matching, resulting in two homogeneous groups with comparable patient, tumor, and surgical characteristics. Nevertheless, MILS remained superior in terms of surgery duration, intraoperative blood loss, hospital length of stay, overall complications, Clavien-Dindo grade ≥ 3 complications, CCI, and readmission rates (Table 5). Despite excellent outcomes, MILS was associated with a low conversion rate (7.7%), with intraoperative bleeding being the most common reason (39.6% of cases, Table 2), consistent with reported literature [38]. Significant differences were noted in the instruments used for liver resections, with energy devices being preferred in MILS cases (86% vs. 46%, *p* < 0.001) compared to the cavitron ultrasonic surgical aspirator (40.9% vs. 70.4%, *p* < 0.001). The higher use of staplers in MILS cases (30.0% vs. 21.9%, *p* < 0.001) may be center-dependent, influenced by early learning curves and/or low-volume programs. According to the 2014 Morioka consensus conference, no specific energy device has proven superior, and the choice remains based on individual surgeon preference [39]. Notably, the argon plasma coagulator was used in only 12.9% of MILS cases in the BReLLS registry, reflecting the conference’s recommendation against its use due to the risk of gas embolism [40]. Center volume was directly correlated with the proportion of MILS performed. Centers conducting at least 50 resections annually demonstrated not only increased surgical complexity but also better short-term outcomes.

This study has some limitations. First, patients undergoing MILS were likely highly selected, particularly in low-volume centers, making direct comparisons between the two groups challenging. Second, we lack long-term outcome data, which would provide more comprehensive insights into oncological results. Lastly, we could not draw significant conclusions regarding the impact of robotic-assisted surgery, which may increase MILS adoption in complex cases while maintaining excellent outcomes. Nonetheless, the BReLLS registry provides a unique opportunity to evaluate indications, techniques, and outcomes from 1,342 consecutive liver resections.

## Conclusions

MILS has established itself as the preferred option for half of all liver resections, particularly in cases involving cirrhosis, portal hypertension, and benign lesions. Its application is mainly limited by expected technical and oncological complexity and previous abdominal surgery. MILS offers superior short-term outcomes compared to the open approach for both minor and major liver resections.

## Data Availability

No datasets were generated or analysed during the current study.
